# Host Range and Specificity of the Drosophila C Virus

**DOI:** 10.1371/journal.pone.0012421

**Published:** 2010-08-26

**Authors:** Martin Kapun, Viola Nolte, Thomas Flatt, Christian Schlötterer

**Affiliations:** Institut für Populationsgenetik, Vetmeduni Vienna, Vienna, Austria; University of Edinburgh, United Kingdom

## Abstract

**Background:**

The Drosophila C virus (DCV) is a common and well-studied *Drosophila* pathogen. Although natural infections are known from *Drosophila melanogaster* and *D. simulans*, and artificial infections have been reported from several *Drosophila* species and other insects, it remains unclear to date whether DCV infections also occur naturally in other *Drosophila* species.

**Methods/Principal Findings:**

Using reverse transcription PCR, we detected natural infections in six *Drosophila* species, which have not been previously known as natural hosts. By subsequent Sanger sequencing we compared DCV haplotypes among eight *Drosophila* host species. Our data suggest that cross-infections might be frequent both within and among species within the laboratory environment. Moreover, we find that some lines exhibit multiple infections with distinct DCV haplotypes.

**Conclusions:**

Our results suggest that the natural host range of DCV is much broader than previously assumed and that cross-infections might be a common phenomenon in the laboratory, even among different *Drosophila* hosts.

## Introduction

The Drosophila C virus (DCV), first isolated from a French *Drosophila melanogaster* strain in 1972 [1], is one of the best studied *Drosophila* pathogens [2]. In contrast to the closely related Cricket paralysis virus (CrPV), which is highly similar to DCV in terms of viral morphology, genome size, and gene arrangement and which infects hosts in several insect orders [3], [4], the known host range of DCV is much more narrow [2]. DCV naturally infects *D. melanogaster* [5], [6] and *D. simulans* [3], [7] from the *melanogaster* subgroup, but natural infections in other species are unknown to date [8]–[10]. To test for susceptibility to DCV, Jousset [11] artificially infected 15 *Drosophila* species, four other dipterans and two lepidopterans by introducing DCV into the abdominal cavity. In addition to monitoring for increased mortality, the author assayed virus maintenance and multiplication by injecting extracts from all artificially infected species into virus free *D. melanogaster*. Jousset found differences in virus susceptibility that range from highly increased mortality in 12 *Drosophila* species (45 strains) to rapid disappearance of DCV in *Culex pipiens* and *Aedes aegypti*. While this result establishes that DCV can artificially infect other species in the laboratory, it remains unclear whether such infections also occur naturally. Here we report a survey for the presence of the DCV in eight *Drosophila* host species (*D. melanogaster, D. simulans, D. mauritiana, D. pseudoobscura, D. subobscura, D. virilis, D. americana*, and an undefined species of the *D. ananassae* group), whose most recent common ancestor dates back 63 million years [12]. We find that DCV has a much broader natural host range than previously thought.

## Materials and Methods

Prior to RNA extraction, we monitored all *Drosophila* lines (approximately 2000 strains) from our fly stock collection by eye for DCV infection symptoms (dark, elongated dead larvae, black dead pupae) [13]. We observed that flies kept in vials at room temperature for more than three weeks showed stronger symptoms than flies kept in vials for shorter periods of time. This might be due to higher susceptibility to viral infection caused by stressfully high larval densities, deteriorating food quality, or high viral loads in the food caused by the presence of dead corpses that release viral particles. Based on these initial observations, we chose 67 strains (*D. melanogaster*: 39; *D. simulans*: 4; *D. mauritiana*: 3; *D. sechellia*: 1; *D. yakuba*: 2; *D. erecta*: 1; *D. willistoni*: 1; *D.* cf. *ananassae*: 3; *D.* cf. *ananassae*: 1; *D. pseudoobscura*: 3; *D. subobscura*: 3; *D. virilis*: 2; *D. americana*: 3; *D. mojavensis*: 1) for molecular characterization of DCV infections because they displayed strong infection symptoms.

RNA was extracted from 5–10 mated females per line using Trifast® (PEQLAB Biotechnologie GMBH, Erlangen, Germany) following the manufacturer's instructions after homogenizing whole flies with an Ultraturrax disperser (IKA® Werke GmbH & Co. KG, Stauffen, Germany) and resuspending in 20 µl RNAse free water. RNA quality was tested on a 2% non-denaturing agarose gel. cDNA was obtained by incubating 3–11 µl RNA with RevertAid™ H-Minus M-MulV Reverse Transcriptase (Fermentas, Germany) and Primer DCV8 (5′-GAAGCACGATACTTCTTCCAAACC-3′) [14] according to the manufacturer's protocol. PCR reactions were performed in a 20 µl reaction volume containing 10 pmol of primers (forward: DCV7, 5′-AGTATGATTTTGATGCAGTTGAATCTC-3′ and reverse: DCV8, 5′-GAAGCACGATACTTCTTCCAAACC-3′) [14], 2.5 mM MgCl_2_, 0.2 mM nucleotides, 0.5 U Taq polymerase (Solis BioDyne, Tartu, Estonia) and 0.5–3 µl of RT reaction product. A typical PCR consisted of the following steps: initial denaturation for 4′ at 94°C; followed by 34 cycles of denaturation for 40″ at 94°C, annealing for 40″ at 52°C, and elongation for 1′ at 72°C; and a final step of 7′ at 72°C. This primer pair amplifies coding DNA from the open reading frame 1 (ORF 1) of the DCV genome. The amplicon spans a region from position 943 to position 1467 and is located in a genomic part which contains domains of a helicase protein. In total, we amplified DCV fragments from 39 *Drosophila* strains.

To gain insights into the genealogical relationship among DCV haplotypes we used Sanger sequencing. ET terminator sequencing chemistry and protocols (GE Healthcare, Little Chalfont, United Kingdom) were used for cycle sequencing of PCR products from 39 strains. For 12 lines (*D. melanogaster*: 8; *D. mauritiana*: 1; *D. mojavenis:* 1*; D. pseudoobscura:* 1; *D. simulans:* 1) we were not able to produce high quality sequence data and thus excluded them from further analysis. Reaction products were sequenced on a MegaBace 500 sequencer (GE Healthcare, Little Chalfont, United Kingdom). All sequences are deposited at NCBI GenBank. They are accessible through accession numbers GU983877–GU983885, GU983888–GU983894, GU983896–GU983902, GU983905–GU983906, and GU983908–GU983911.

CodonCodeAligner v. 3.5.2 (http://www.codoncode.com/aligner/download.htm) was used for editing raw electropherograms, assembly of contigs, and for alignment of sequences. To avoid errors caused by lower sequence quality in the proximity of primers in some samples, we restricted our analysis to a 500 bp region that was sequenced in both directions for most of the samples (see File S1). Missing nucleotides due to low sequence quality at the fragment ends of some samples were filled with Ns (<18% of the sequence) to avoid loss of information due to clipping of the whole sequence alignment. Lines that contained heterozygous sites (sites with two overlapping peaks) in the electropherogram at the same position in the forward and reverse sequence were considered to contain more than one viral haplotype. Since consistent differences in peak height on both strands likely reflect variation in the abundance of viral haplotyes, we used this information to infer viral haplotypes. Thus, all sequence variants with low peaks were attributed to one, whereas variants with high peaks were attributed to another haplotype (see Figure S1).

Using jModelTest [15] we found GTR+Γ+I [16] to be the best fitting substitution model. PhyML [17] was used to calculate an unrooted maximum likelihood tree using the GTR+Γ+I model with eight discrete rate categories for the Γ-distribution. We additionally performed a likelihood ratio test to compare the GTR+I and the GTR+Γ+I models. MacClade version 4.06 [18] was used to estimate the number of character changes along the topology of the maximum likelihood tree as calculated with PhyML. The tree was plotted and edited using Figtree version 1.3.1 (http://tree.bio.ed.ac.uk/software/figtree/). To test for recombination we performed a Phi-Test [19] and used GARD [20] from the datamonkey website (www.datamonkey.org).

## Results and Discussion

Using RT-PCR we found natural DCV infections in eight different hosts (*D. melanogaster, D. simulans, D. mauritiana, D. pseudoobscura, D. subobscura, D. virilis, D. americana*, and an undefined species of the *D. ananassae* group). Two of these species (*D. simulans*, *D. melanogaster*) are known to be both artificial and natural hosts of DCV [3], [5]–[7], [11], and our results confirm that natural infections occur in these species. We also identified two novel natural hosts (*D. mauritiana, D. virilis*) for which only artificial infections have been reported so far [11]. In addition, we found evidence for four natural hosts (*D. pseudoobscura, D. subobscura, D. americana*, *D.* cf. *ananassae*) which have neither been reported as natural nor artificial hosts before [8]–[10]. Our results thus suggest that DCV is naturally infecting a much broader range of *Drosophila* host species than previously thought.

**Figure 1:**
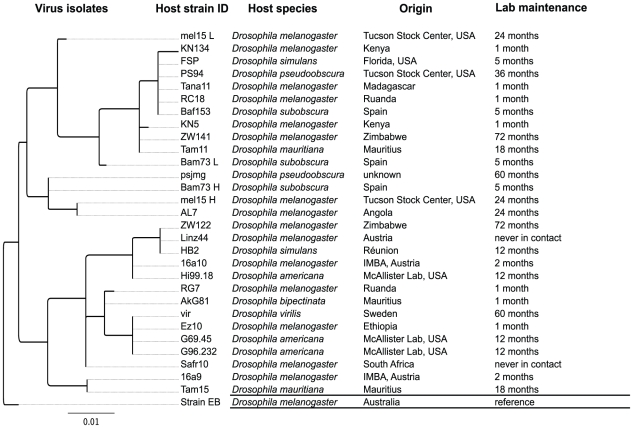
Genealogy of DCV isolates. Unrooted maximum likelihood tree showing all virus samples identified in our dataset (see File S1), including host identity (ID), host species, last known origin of the sample, and duration of laboratory maintenance in our stock collection until the time of RNA extraction. Host lines “never in contact” are wild caught lines which have never been in physical contact with lab stocks; all other lines are lab stocks. Host ID contains information about whether strains were extracted from multiply infected samples (L: low peaks; H: high peaks; see Materials and Methods and File S2).

We sequenced 500 bases of the PCR fragment to shed more light onto the origin and diversity of the DCV samples detected in the eight *Drosophila* species. In 28 samples (including the isolate EB: GenBank accession number NC_001834) we identified 16 distinct haplotypes (Figure 1). In two samples we detected more than a single DCV haplotype. Although infections with multiple DCV isolates have been described before [21], [22], we found that multiple infections do not only occur in *D. melanogaster*, but also in *D. subobscura*. As expected from the high haplotype diversity, most of the DCV haplotypes in strains with multiple infections were unique. Nevertheless, one DCV haplotype was also detected in other strains. This observation strongly suggests that in a given strain multiple DCV haplotypes are the outcome of several infections and not due to mutations in the host.

To infer the genealogical relationship among the DCV haplotypes, we performed a phylogenetic analysis. Based on the inferred tree, we did not observe any clustering of haplotypes according to their host species (Figure 1), suggesting that DCV lacks host specificity. Under the assumption of common ancestry of the sequences obtained from DCV isolates in our laboratory and the EB isolate deposited in GenBank 11 years ago [23], we estimated an upper bound of the mutation rate (i.e., the number of variable synonymous sites divided by the sequence length times divergence time). We found 3–10 synonymous substitutions between our sequences and the EB isolate (mean: 6.5; standard deviation: 2.33), suggesting an approximate mutation rate/(site x year) of 5.5×10^−4^ to 1.8×10^−3^. These values are comparable to estimates for other RNA viruses [24], [25], for example for Sigma virus (1.0×10^−4^ [26]), and human Influenza A (2.6×10^−3^) and B (5×10^−4^ [27]).

Since in our study several DCV isolates were separated from each other by more mutations than from the published isolate, the date of divergence of DCV isolates identified in our laboratory may be similar to the time of split from the EB isolate. Thus, the divergence observed among the DCV isolates might predate the arrival of these isolates in our laboratory and is unlikely be due to recent mutations. Nonetheless, the absence of host specificity and spatial structure in our data strongly suggests that the isolates we detected are lab-specific epidemics, for example originating from cross-infections with previously infected lab strains. Interestingly, Johnson and Christian [22] found geographical clustering among DCV haplotypes in *D. melanogaster* using PCR-RFLP, but we failed to find any clustering of our samples with respect to their geographical origin (see Figure 1). However, since some of our fly strains have been maintained as lab stocks for a considerable amount of time, we cannot rule out that existing geographic patterns have been erased by cross-infections in our laboratory, a possibility also raised by Johnson and Christian [22].

Since high rate heterogeneities may cause high rates of homoplasy, thus obscuring the true genetic distance, we estimated α, the shape parameter of the Γ-distribution. A likelihood ratio test based on the GTR and GTR+Γ models showed that using the Γ-distribution significantly improved the fit of the model to our dataset (p<0.001, 2ΔlnL =  27.2504, χ^2^_0.001,df = 1_ = 10.83). Using PhyML we obtained a shape parameter α of 0.101. An α-value as low as 0.1 yields a sharp L-shaped distribution, with many sites being virtually invariable and with few sites that exhibit very high mutation rates [28].

**Figure 2:**
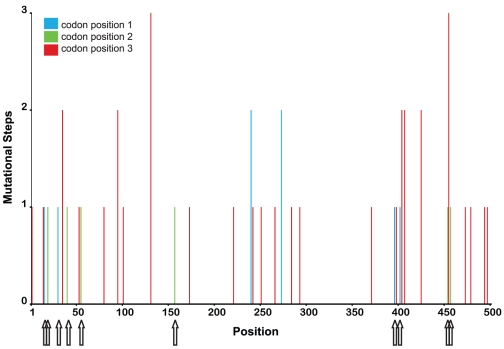
Minimum mutational steps. Minimum number of mutational steps per site estimated by MacClade. The arrows indicate mutations which led to amino acid changes.

To gain further insight into the distribution of recurrent mutations we used parsimony to estimate the minimum number of mutational steps at each site, based on the inferred phylogenetic tree. Consistent with our maximum likelihood analysis, we found a large number of invariant sites, in combination with highly variable sites (see Figure 2). Interestingly, the highly mutable sites were distributed over the entire sequence and mainly affected the third codon position. Four amino acid replacements were inferred to be recurrent mutations (see Figure 2). While these data suggest that the divergence between the sequenced DCV isolate and our isolates might be due to a high rate of homoplasy not adequately recovered by maximum likelihood, we note that recombination, which is a well-described phenomenon among RNA viruses [29], also could have generated this pattern. To test this possibility we performed a PHI-Test (p>0.05) and GARD analysis. Both tests did not provide any evidence for recombination. Thus, within the limitations of the statistical power of tests for recombination, our analyses suggest that homoplasies found in our dataset result from recurrent mutations.

To investigate whether the presence of different isolates in a multiply infected host strain is changing over time, we propagated two *Drosophila* lines (*D. melanogaster, D. mauritiana*) for three months and compared the DCV sequences at the beginning and the end of this period. For both lines, we observed identical sequences.

Since it is possible that DCV infection is mainly a laboratory phenomenon, we also inspected offspring of freshly collected wild-type *D. melanogaster* lines that have never been in contact with the laboratory environment. Out of 420 *D. melanogaster* lines, we selected two that showed clear DCV infection symptoms (see Material and Methods). RT-PCR confirmed infection of these lines with DCV. We found that one isolate represents a new haplotype, whereas the other was identical to one of our laboratory isolates. However, as a cautionary note, it must be mentioned that the flies were collected by a researcher with continuous exposure to laboratory *D. melanogaster*.

In summary, our data show that DCV can infect a much broader range of *Drosophila* species than previously reported. Due to the total absence of spatial structure and host specificity in our data, we conclude that there is a high degree of cross-infection with DCV among laboratory strains of the genus *Drosophila*. Since DCV is transmitted horizontally [1], [30], [31], laboratory maintenance might in fact facilitate cross-infections among different *Drosophila* lines and species that are kept in culture. Although this might be a potential nuisance for researchers working with multiple species, it also raises interesting questions. One such question is whether distinct host species co-evolve differently with DCV. For example, since the disease symptoms of DCV infection have so far only been characterized in *D. melanogaster*, it would be interesting to study in future work the pathophysiology of this virus in other *Drosophila* species that we have identified here as natural hosts. Moreover, since DCV is known to influence a number of physiological and life history traits (e.g., mortality, fecundity and body size) in *D. melanogaster* [30], [32]–[34], future studies should take into account that DCV might have large and potentially confounding effects on such traits in a wide range of host species.

## Supporting Information

File S1 Raw electropherograms of sequenced DCV isolates.
(1.72 MB ZIP)

File S2 FASTA alignment of consensus sequences from DCV isolates.
(0.02 MB TXT)

Figure S1 Electropherograms from sample Bam73, which contains more than one virus isolate (Top row: forward sequence; bottom row: reverse sequence). The arrows indicate three positions with polymorphisms. The different signal intensity of the variable sites allowed us to infer the two haplotypes. We determined C,T and T to belong to the high frequency haplotype and T,C and C to the low frequency haplotype.
(0.83 MB EPS)
